# MedLingua: A conceptual framework for a multilingual medical conversational agent

**DOI:** 10.1016/j.mex.2024.102614

**Published:** 2024-02-22

**Authors:** Galib Muhammad Shahriar Himel, Md. Shourov Hasan, Umme Sadia Salsabil, Md. Masudul Islam

**Affiliations:** a Department of Computer Science, American International University-Bangladesh, Dhaka, Bangladesh; b School of Computer Sciences, Universiti Sains Malaysia, 11800 USM Penang, Malaysia; c Department of Computer Science and Engineering, Bangladesh University of Business and Technology, Dhaka, Bangladesh

**Keywords:** Medical chatbot model, Expert system framework, Medical assistant model, System design, Conceptual architecture, A Scrum-Based Methodology for Dynamic Medical Chatbot Solutions

## Abstract

This study introduces a hybrid model for an advanced medical chatbot addressing crucial healthcare communication challenges. Leveraging a hybrid ML model, the chatbot aims to provide accurate and prompt responses to users' health-related queries. The proposed model will overcome limitations observed in previous medical chatbots by integrating a dual-stemming approach, P-Stemmer and NLTK-Stemmer, accommodating both semitic and non-semitic languages. The system prioritizes the analysis of cognates, identification of symptoms, doctor recommendations, and prescription generation. It integrates an automatic translation module to facilitate a smooth multilingual diagnostic experience. Following the Scrum methodology for agile development, the framework ensures adaptability to evolving research needs and stays current with recent medical discoveries. This groundbreaking idea aims to improve the effectiveness and availability of healthcare services by introducing an intelligent, multilingual chatbot. This technology enables patients to communicate with doctors from diverse linguistic backgrounds through an automated language translation model, eliminating language barriers and extending healthcare access to rural regions worldwide.

• A simple but efficient hybrid conceptual model for advancement in smart medical assistance.
• This conceptual model can be applied to implement a medical chatbot that can understand multiple languages.
• This method can be utilized to address medical chatbot limitations and enhance accuracy in response generation.

## Graphical abstract

**Figure:**
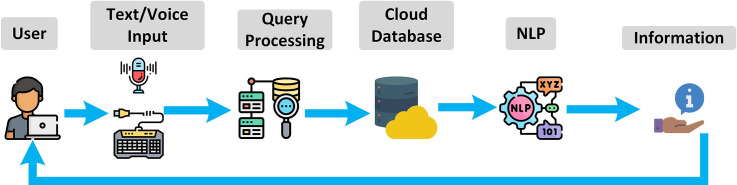

Specifications Table

**Table:** 

Subject area:	Computer Science
More specific subject area:	System Design, Framework Architecture, Conceptual Architecture
Name of your method:	A Scrum-Based Methodology for Dynamic Medical Chatbot Solutions
Name and reference of original method:	Not Applicable
Resource availability:	Not Applicable

## Method details

In the global health sector, ensuring people's access to healthcare, even if it involves basic services, has consistently been a primary focus. According to a Lancet study [1], an estimated 5 million individuals die annually due to substandard healthcare in low- and middle-income countries. This figure surpasses the 3.6 million deaths in these nations resulting from a lack of healthcare access. In low- and middle-income countries, which constitute the majority of the world's 134 nations, there are 8.6 million annual deaths that could have been prevented with effective healthcare systems. These fatalities were attributed to treatable conditions resulting from inadequate care. Approximately one-third of patients consistently report subpar experiences, characterized by disrespectful treatment, excessively brief consultations, poor communication, and prolonged waiting times. Shockingly, a significant percentage of parents, ranging from 40 to 50 percent, leave medical facilities without knowledge of their child's diagnosis.

A medical chatbot can significantly address the challenges outlined above by providing accessible and efficient healthcare services. Firstly, it ensures immediate access to basic healthcare information, offering users a quick response to their queries. By leveraging advanced algorithms and medical databases, the chatbot can aid in preliminary diagnoses and guide preventive care. This promotes early intervention and potentially reduces the number of deaths attributed to poor-quality healthcare. Moreover, the chatbot's continuous availability minimizes the issue of short visits and long wait times, offering users a consistent and prompt healthcare experience. Through clear and concise communication, the chatbot can enhance patient understanding of medical conditions and treatment plans, reducing the percentage of individuals leaving without a proper diagnosis. Overall, the implementation of a well-designed medical chatbot can contribute to improving the quality of healthcare services, addressing key issues, and ultimately saving lives, especially in low- and middle-income countries.

Medical chatbots, if effectively implemented, hold significant value for healthcare providers by reducing costs and enhancing administrative efficiency. Recent studies indicate an 87.58% satisfaction rate. Offering voice and text interaction, chatbots provide immediate responses, minimizing waiting times, and increasing customer satisfaction. Addressing limitations in existing systems, the proposed medical healthcare chatbot leverages artificial intelligence, introducing specifically trained models and AI-driven solutions to improve accuracy, prevent misdiagnoses, and offer instant problem-solving, ultimately saving patients costs associated with unnecessary doctor consultations.

Several related works have been done previously such as, the importance of linguistic interaction in counseling is highlighted, utilizing Natural Language Processing (NLP) and Natural Language Generation (NLG). Oh et al. [2] in the year 2017 did a research on applying AI algorithms to recognize and classify emotions. The studies use recurrent neural networks (RNNs), deep learning, and convolutional neural networks to train emotions classification models from a large amount of labeled data. Linguistic interaction is crucial in counseling, which relies on Natural Language Processing (NLP) and Natural Language Generation (NLG) to comprehend user discussion. Emotion recognition is approached via a multi-modal strategy in this case. They collected corpora to learn semantic information about words and express it as a vector using the word vector, as well as lexical synonym knowledge.

du Preez et al. [3] develops a voice recognition chatbot. The bot's questions that are not comprehended are sent to a third-party expert system for further processing. Web-bots are produced as text-based web-friends that entertain users. They concentrated on the enhanced system here if the application is not only text-based but also voice-based. For voice recognition, a two-step procedure of recording and analyzing an input signal is necessary. Data from the server response is recognized and information is processed. The server utilized here is a SOAP-based black box technique. It is feasible to improve unlimited and autono-mous intelligence by using an expert system.

Setiaji and Wibowo [4] developed a chatbot to managed human-machine interaction. The system maintains the knowledge database in this location to identify the sentence and decide on how to respond to the question. The similarity score of input sentences will be calculated using bigram for the input sentence. The knowledge of the chatbot is maintained in a relational database management system (RDBMS).

Dahiya [5] developed a chatbot which uses pattern comparison to recognize and save the sentence order. The author explains how the chatbot's operating system, software, programming language, database, and input and output results are stored in this article. The text method is used to extract the input, and the trim function is used to re-move other punctuation. The random function is then used to select a response from the database. The chatbot serves as a source of amusement.

Shabariram et al. [6] use the n-gram technique for extracting the words from the sentences. With Moro phonemes and phonemes as the determining parameter, annagram is utilized to compare and deduce the input with case data. For the closest match, probability analysis is carried out. The final expression is redirected through an expert system.

For the Android application, a chatbot was created for healthcare purposes in the year 2018. The user uses Google API to send a text or voice message. The user receives only related responses from the chatbot in this case. The dataset is classified using the SVM method. The Porter method is used to filter out words that are not needed, such as suffixes and prefixes [7].

Kondrak and Dorr [8] discussed how to reduce medical errors caused by drug names that are similar but not identical. The method uses two new methodologies, one based on orthographic similarity (“look-alike”) and the other on phonetic similarity (“sound-alike”). For determining the efficacy of different similarity measures on medicine names, a new recall-based evaluation methodology is given. On a set containing both look-alike and sound-alike pairs, the novel orthographic measure (BI-SIM) surpasses other commonly used similarity measures, while on a test set containing exclusively sound-alike confusion pairs, the feature-based phonetic approach (ALINE) beats orthographic approaches. However, for both test sets, a technique that combines numerous different variables yields the greatest results.

Kondrak and Sherif [9] assessed the challenges to manage phonetic similarity with a focus on the identification of cognates, or words with the same origin in different languages. To compute phonetic similarity, representatives of two main methodologies were compared: manually-designed measures and learning algorithms. A stochastic transducer, a Pair HMM, multiple DBN models, and two built schemes were all considered. Those techniques were evaluated both supervised and unsupervised on the job of finding cognates among Indo-European languages. Given a large training set of positive instances, the averaged context DBN model and the Pair HMM appear to have the highest accuracy.

Nagai et al. [10] attempts to determine the demands and challenges of using a care robot in a nursing care context. As a result, there is a scarcity of information about the effects of the introduction of care robots, the demands of patients and caregivers, and ethical issues that may arise. There are numerous things to consider progressing research and incorporating care robots into settings, including investigating, extracting needs, clarifying ethical issues, and seeking answers, conducting an on-site experiment study, and so on.

Mackay and Kondrak [11] invented a technique which described the calculation method of assessing the similarity of two words. The technique is based on Pair Hidden Markov Models, a kind of Hidden Markov Model that has been effectively employed for biological sequence alignment. The model's parameters are learnt automatically using training data, which consists of word pairings that are known to be related. These tests are focused on identifying cognates, or words that have a shared origin in languages that are related. This approach outperforms previously presented strategies, according on the results.

Martin et al. [12] developed ‘Symptoma’ which is a symptom-to-disease digital health assistant that can distinguish over 20,000 illnesses with a 90 percent accuracy rate. Using a variety of clinical situations and COVID-19 case reports, they investigated the accuracy of Symptoma in identifying COVID-19. In 96.32 percent of clinical cases, they found that Symptoma can reliably detect COVID-19. When just COVID-19 symptoms and risk factors were in-cluded, Symptoma correctly diagnosed 100 percent of individuals affected when only three indicators were provided. Finally, researchers demonstrated that the accuracy of Symptoma considerably outper-forms that of basic “yes–no” surveys commonly available online. Symptoma has unrivaled accuracy when it comes to methodically diagnosing instances of COVID-19 while simultaneously taking into account over 20,000 other disorders. In addition, Symptoma supports free text input in 36 languages, which is supplemented by disease-specific follow-up questions. Symptoma has the potential to be a crucial instrument in the global fight against COVID-19, based on its findings and accessibility.

Miner et al. [13] disscused the usage of chatbot to fight COVID-19 along with a comparison of different healthcare related chatbots in his research done in the year 2020.

Srivastava and Singh [14] created a diagnosis chatbot to engage patients in a discussion about their medical questions and difficulties in order to deliver a personalized diagnosis based on their diagnosed symptom and profile. With a standard precision of 65 percent, the chatbot system is qualified to diagnose symptoms from user inputs. Correct symptoms were recognized with a recall of 65 percent and precision of 71 percent using these extracted diagnostic symptoms. Finally, the chatbot provided the anticipated diagnostic for more procedures. This shows that a medical chatbot may offer a patient with a reasonably correct diagnosis using simple symptom analysis and a conversational approach, implying that a practical spoken language medical bot is possible. Furthermore, the bot's relative performance suggests that as time goes on, more auto-mated medical goods may grow, allowing them to play a larger part in healthcare.

Mathew et al. [15] suggested a system that was designed to provide an alternative to the traditional practice of visiting a hospital and scheduling a doctor's appointment to receive a diagnosis. The goal of this study is to construct a chatbot application using natural language processing and machine learning technologies. People may communicate with the chatbot in the same way they would with another human, and the chatbot will recognize the user's symptoms and, as a result, anticipate the condition and prescribe therapy through a series of questions. This technique may be very useful in doing daily check-ups, mak-ing individuals aware of their health state, and encouraging them to take the necessary precautions to stay healthy.

Rosruen and Samanchuen [16] created a chatbot system using data from the DoctorMe program, which included symptoms and treatment histories. The test results demonstrate the suggested system's capacity. Furthermore, it may be utilized as a guideline for future enhancement as well as future research.

Gupta et al. [17] created a chatbot to assist customers in predicting their potential sickness through a simple discussion in which they would be questioned about their symptoms, emotions, and diets. It will inform indi-viduals about the severity of their sickness and whether or not they need to take action to combat it.

Hwang et al. [18] developed interactive healthcare advisor model using chatbot and visualization. The components required for establishing the interactive healthcare advi-sor model (IHAM) and chatbot-based IHAM were detailed in their paper. Body temperature, oxygen satu-ration (SpO2), pulse, electrocardiogram (ECG), and other biological information of target users were measured and analyzed with biological sensors based on the oneM2M platform, as well as employing an interactive chatbot to assess common biophysical circumstances. Furthermore, the chatbot delivers to users the gathered biological information in the chatbot and the biological sensors, as well as medical advice to improve the user's overall health. They will confirm the applicability of the health care advisor model in the future and provide future research directions as a result of its deployment.

Astuti et al. [19] developed a chatbot COVID-19 cases. The RASA framework is used in this study to predict chatbot replies to questions regarding COVID-19, and the DIET Classifier is used for training. The test results using the DIET Classifier model is around 85%.

Comparative studies of various medical or healthcare chatbots are summarized in Table 1.

**Table 1:** Comparative studies of various medical chatbot system.

Study	Methodologies	Functionality	Outcome	Pros	Cons
Shabariram et al. [6]	n-gram is used, Moro phonemes and phonemes are used as determining parameter	Android-based expert system for user interaction	User receive general suggestion regarding dieases	Smartphone App is user friendly and easy to understand	Cannot provide detailed prescription regarding diseases
Martin et al. [12]	A prediction engine uses the references from symptom- diseases database curated by medical doctors	Assessing the input text it can identify specific COVID-19 severity, can distinguish other flues	Detecting COVID-19 cases with a 96.32% accuracy in clinical cases	Rapid identification of COVID-19	Other diseases are not focused upon
Srivastava and Singh [14]	Pattern recognition using Artificial Intelligence Mark-up Language which is based on XML	Android-based interface for HCI	Analysing the symptoms given in the input panel it predicts the diseases with a precision of 65% and gives prescription accordingly	Follows a conversational approached using commonly spoken language	The precision rate is relatively lower
Astuti et al. [19]	DIET classifier is used along with RASA framework	This chatbot answers questions related to COVID-19	This chatbot achieves 85% accuracy	The model generation was successful without data normalization	This chatbot is only focused on COVID-19 cases
Divya et al. [20]	It provides a Linear Design encompassing elements such as Artificial Intelligence, Pattern Matching, Disease, and Query Processing techniques. The system offers a finite state graph for chatbot dialogues along with a functional architecture.	Recognizes the relevant symptom, associates it with either a minor or major ailment, and offers guidance based on the identified condition.	Offers individualized diagnoses according to presented symptoms	It offers a user-friendly interface, and the integration of the chatbot can enhance the efficiency of delivering improved treatment within a brief timeframe.	The chatbot recognizes both minor and major illnesses, guiding whether it is advisable to consult a doctor or not.
Kosarkar et al. [21]	It presents a diagram illustrating the sequence of disease prediction, including datasets, feature selection, data preprocessing, decision tree classification, text processing, and keyword matching.	Using the symptom dataset, the system accurately forecasts the presence of the disease.	Convenient to reach and facilitates instant communication through contemporary and up-to-date technologies.	Decision tree algorithms are employed to enhance the precision of disease prediction.	The chatbot might encounter difficulties in effectively managing any novel symptoms presented by a patient.
Vaira et al. [22]	Support Vector Machine (SVM) algorithm, Porter Stemming algorithm, and word similarity	Details regarding medication and recommended dosage. Prediction of diseases.	Four datasets were employed to provide information on heart disease across cities of varying sizes. The classifier training involved 60% of each dataset, while the remaining 40% was allocated for testing purposes. Cleveland exhibited the highest training count among the cities. Three algorithms—KNN, Naïve Bayes, and SVM—were tested, with SVM demonstrating superior accuracy.	It is feasible to engage in conversations using voice and text interchangeably.	Lacks navigation capability.
Lalwani et al. [23]	Lemmatization and part-of-speech (POS) tagging, evaluating semantic sentence similarity, and the development of artificial intelligence modeling language (AIML).	Accessing user profiles to retrieve attendance records and grade/point data. Reviewing placement details. Supplying comprehensive information related to admissions.	Not mentioned	Once the user is authenticated, their profile is presented.	Conversations involving voice and text are not achievable.
Harkous et al. [24]	Personalised privacy policies	Providing privacy statements and revising the policies.	Offers a more engaging method of presenting policies to the user and empowers the user with greater control.	Maintaining user trust in a chatbot is upheld by having adaptable privacy policies.	Some policies may lack flexibility.
Suffian et al. [25]	It provides the K-means algorithm, the LAMSTAR network, the compilation of medical datasets, and the comparison of symptoms through an iterative search.	Its primary emphasis lies in the text processing and feature extraction domains. Through classification, it provides recommendations for disease prevention.	In this assessment, outcomes are assessed using precision, recall, and F1-scores related to diseases, and based on the achieved accuracies, naive Bayes demonstrates inferior performance compared to Random Forest and Decision Tree.	It has computed outcomes for various keywords and key phrases, achieving positive results with limited datasets.	In this particular real-time and context-specific scenario, datasets about local issues have not been acknowledged. Instead, the analysis relies on limited datasets.

Analyzing the previous medical-related chatbots and related knowledge gaps, we are proposing a new framework for an advanced medical chatbot. In this development, we are going to follow a methodology supported by the Scrum Framework. For development purposes, Waterfall, Scrum, and DSDM are the most popular software development methodologies. Among them, the Waterfall method is meant for long development but the modification process cannot be done in the middle of development. Also, new features cannot be added in the development phase if not mentioned in advance. But, in this kind of research-based development, new ideas may pop up at any time. That is why the Waterfall method is not suitable for our research.

On the other hand, Scrum allows sudden changes while in the middle of development which is exactly what we need in our research project. Also, Scrum tasks are divided into sections that can be executed independently, which is of immense help in research projects. That is why we will use the Scrum methodology for our development. In case of any modification, we will change the Sprint distribution accordingly. Also, a benefit of Sprint is the actual experiment or development is divided into independent working segments. That's why if anything goes wrong in any particular application segment, that can be fixed easily without much hassle. Following the above framework, the database will be created and updated continuously, and at the same time, the Machine Learning models will be upgraded continuously.

But, to create the ML training Models, various ML algorithms are to be researched thoroughly. Convolutional Neural Networks (CNNs) are powerful in computer vision, detecting patterns like lines and faces in raw images. Support Vector Machines (SVMs) excel in classification and regression, creating well-separated data maps. Naïve Bayes Classifiers utilize Bayes' Theorem, assuming independence between categorized characteristics. The Random Forest Algorithm, adept at handling diverse datasets, employs decision trees for classification and regression. While other algorithms exist, the proposed system emphasizes CNN, SVM, Naïve Bayes, and Random Forest for optimal performance.

### Proposed system

The aim is to define a chatbot framework that will take both voice and text commands as input and handle the cognate-related problems of the medical field with special emphasis on chemical and drug names. The objectives are given below:

• To provide an efficient solution to a necessary part of the healthcare process.
• To build an informative database for each user according to symptoms, treatment, and timeline.
• To identify cognates correctly to avoid misinterpretation.
• To provide an option in case of confused/mispronounced/misspelled words to make sure the processing of diagnosis is impeccable.
• Scheduling and reminding appointments and medicine intake.
• To develop multi-lingual features.
• To develop auto-translation features.
• To handle medical terms and cognates related to drug names/chemicals specifically using different medical databases.
• To provide emotional support through psychology.

**Fig. 1:**
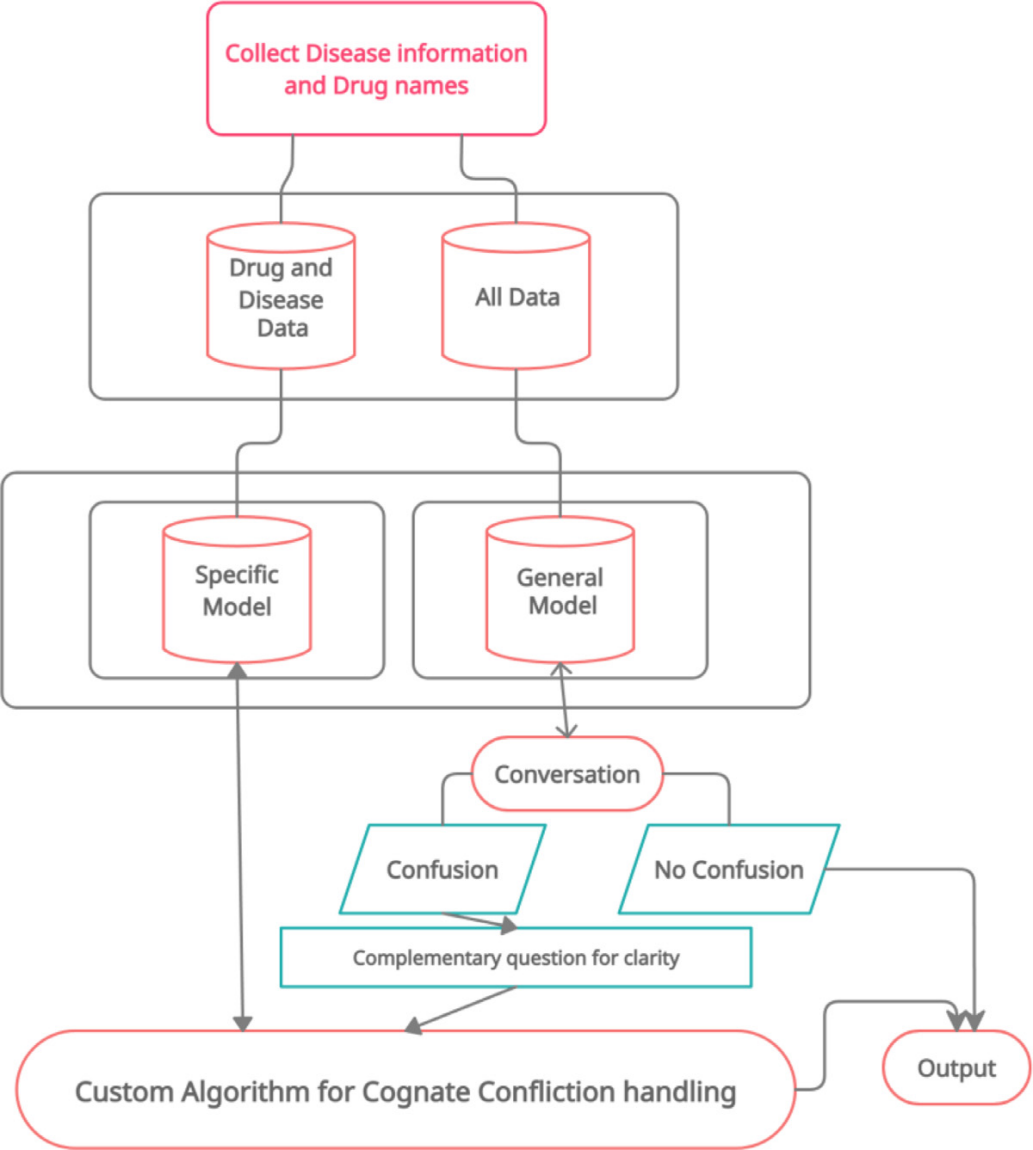
System flowchart.

Fig. 1 illustrates our proposed system flowchart. The details of the proposed system are described below:

• Users can chat with the bot through both voice and text.
• The system will use an expert AI-based bot to answer the queries.
• Users can also view the available doctors for that specific disease.
• Every conversation will be stored in the form of a pattern template.
• Bot will provide analgesics, foods & and medicine suggestions which means which food or medicine you must take based on the disease.
• Complementary question in case of cognates confliction.
• Specific search after ensuring the exact words.

**Fig. 2:**
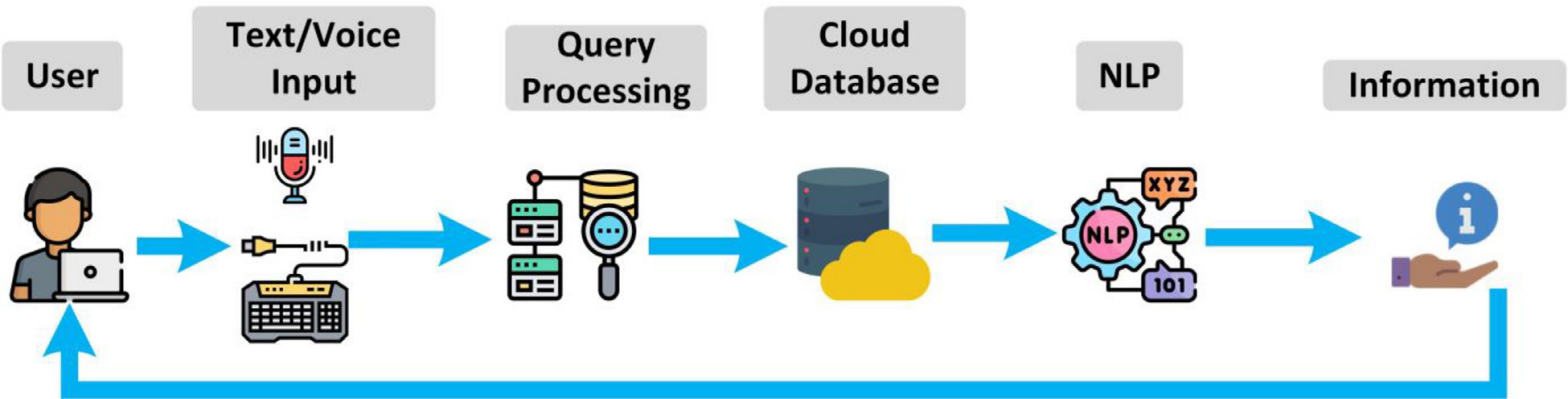
Proposed system architecture.

For implementing a well-planned system, a workflow model describing the system architecture is necessary. Our proposed system architecture is shown in Fig. 2.

### System implementation

The entire system is based on Artificial Intelligence. To implement the system properly, a Machine learning algorithm is necessary. However, there is a plethora of algorithms for implementing ML. This paper demonstrates a hybrid ML-based framework consisting of Four ML algorithms named CNN, SVM, B, and RF. These four algorithms are reused for training and then the voting and averaging function will be used to get the best output. In the context of voting, there are two approaches: Hard voting and Soft voting, which can be represented by Eqs. (1) and (2). With hard voting, the class with the highest number of votes, denoted as N_c_(y_t_), is selected. This involves predicting the class label **y ^** through the majority vote of each classifier. On the other hand, soft voting entails summing up and averaging the probability vectors for each predicted class from all classifiers. The winning class is determined by the highest value, a method advisable when the classifiers are well-calibrated.

(1) $$\hat{y}=argmax\left(N_{c}\left(y_{t}^{1}\right),\;N_{c}\left(y_{t}^{2}\right),\;\ldots \ldots \ldots \;N_{c}\left(y_{t}^{n}\right)\right)$$

(2) $$\hat{y}=argmax\left(\frac{1}{N_{Classifiers}}\right)\sum_{Classifiers}\left(P_{1,\;}P_{2},\;P_{3}\ldots \ldots \ldots \;P_{n}\right)$$

Also, Eq. (3) represents the formula for averaging. Where n is the number of classes and $AP_{k}$ is the average precision of class ***k***

(3) $$Avg=\;\frac{1}{n}\sum_{k=1}^{k=n}AP_{k}\;$$

However, before training, P-Stemmer and NLTK-Stemmer will be used to do the stemming as classification is based on the stemmed/root word. After collecting data, every sentence will have to be divided into words and then the steaming process will be done to get root words after that, the training of the model, and classification of the result generations will be completed.

**Fig. 3:**
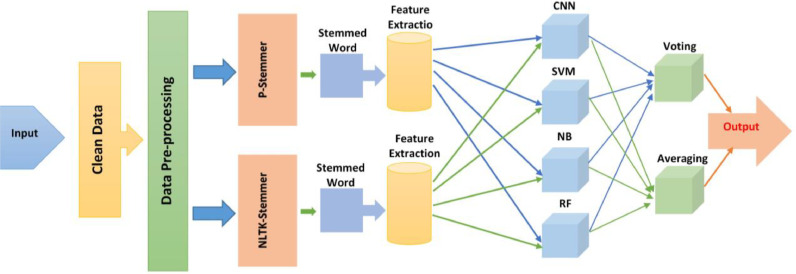
Proposed hybrid framework.

Two different stemmers will have to be used in this proposed model. Since the stemming process of semitic and non-semitic languages are different a single stemmer is not sufficient for extracting features related to root words from all languages. P-stemmer is well known for extracting root words from Semitic languages, specifically Arabic. On the other hand, NLTK is used for extracting root words from non-semitic languages. Though it works better for those languages that are based on Latin origin, it also can be used in other non-Semitic languages with some modifications. That's why both stemmers are needed for a comprehensive machine learning model to be built which will be able to deal with all renowned languages. The proposed hybrid framework is presented in Fig. 3.

**Fig. 4:**
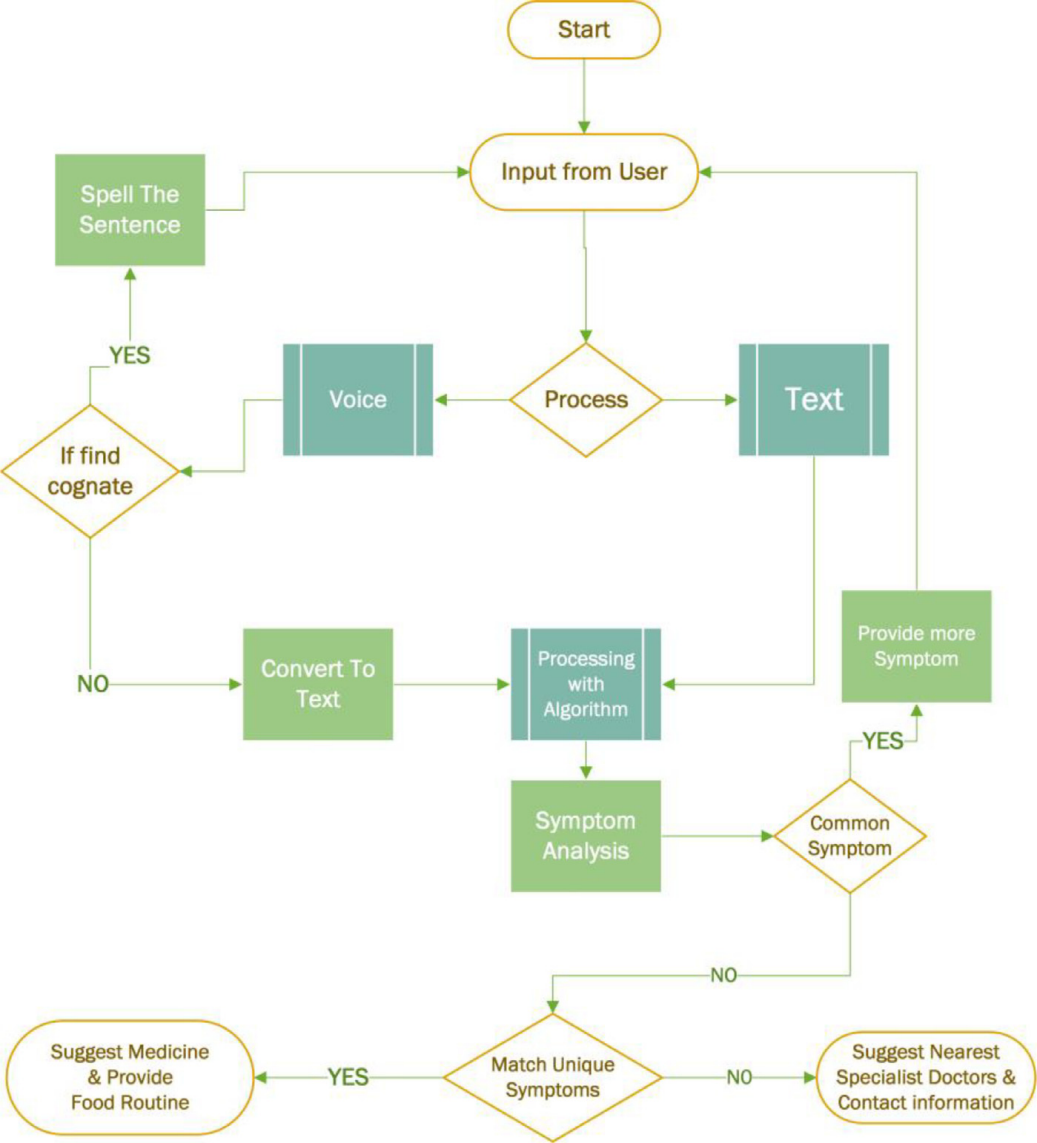
Dataflow diagram of the proposed chatbot.

To handle the conversation with the chatbot the process described in Fig. 4 will be followed.

The dataflow diagram described in Fig. 4. can be represented as an algorithm as follows:

**Table:** Algorithm 1

**Step 1**. INPUT: Text OR Speech
**Step 2.***If* Text *Then*
*Directly pass the input to the next step *
*Else*
*If* Cognate is **True***Then*
*Spell the Sentence*
*Go To:***Step 1**
*Else*
*Convert to Text*
*End If*
*End If*
**Step 3.***If* common symptoms *Then*
Ask for details
*Go To:***Step 1**
*Else*
Match unique symptom
*End If*
**Step 4**. *If* Unique Symptom != Database Stored Information *Then*
Suggest relevant doctors
*Else*
Provide prescriptions and food routine
*End If*

Algorithm 1 describes the general process. That is why the details voice to voice-to-text conversion are not mentioned in the algorithm section. To handle the voice recognition, the *SpeechRecognition* python library which includes several speech recognition engines such as; Google Cloud Speech API, IBM Speech To Text, Microsoft Bing Voice Recognition, CMU Sphinx, etc., or even any custom API can be used in the backend.

**Fig. 5:**
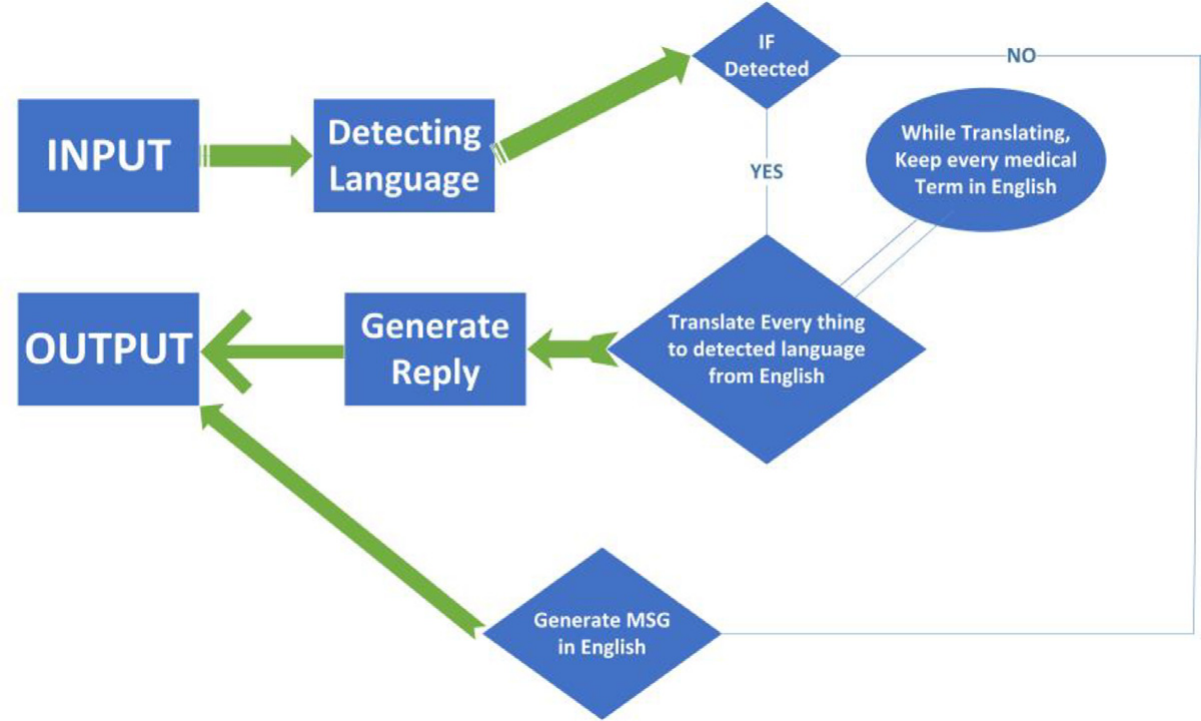
Language detection and automatic translation module.

In case of a doctor's suggestion, the bot will check the location of the customer/patient and suggest the nearest doctor. Otherwise, the bot will provide detailed medical prescriptions along with proper diet, and at the same time, a routine maintenance notification system will be activated. However, the automatic translation process is not included in the data flow diagram. The chatbot system will also use a language detection module. When a user gives input, the bot will analyze the phonetics/syntax and after detecting the language, it will answer the query in the customer's language. A concise overview of Machine translation and automatic language detection is shown in the following Fig. 5.

This whole language module will work at the backend so this figure is described separately to describe this particular task in a better way. If the module can not detect any language, then, the module will ask the user in English to communicate in English. Otherwise, any detectable language will be successfully translated.

## Conclusion

This paper introduces the transformative potential for the healthcare landscape. By addressing the limitations of existing medical chatbots, our work aims to revolutionize patient-doctor interactions. The incorporation of a specific trained model in our chatbot, as opposed to relying solely on conventional databases, will enhance accuracy and ensure that users receive precise information tailored to their medical concerns. Furthermore, our emphasis on leveraging artificial intelligence to recognize various diseases and provide tailored responses will mitigate the risks associated with misdiagnosis. This approach not only contributes to immediate problem resolution but will also alleviate the financial burden on patients who would otherwise only seek consultation for major issues. Additionally, the introduction of a multilingual interface with an automatic translation module opens up new frontiers in healthcare accessibility, enabling individuals from diverse linguistic backgrounds to engage seamlessly with healthcare providers. The implications of our research extend beyond mere technological innovation, promising to foster a more efficient, inclusive, and patient-centric healthcare ecosystem.

## CRediT authorship contribution statement

**Galib Muhammad Shahriar Himel:** Conceptualization, Methodology, Investigation, Resources, Writing – original draft, Writing – review & editing, Visualization, Supervision, Project administration. **Md. Shourov Hasan:** Conceptualization, Methodology, Investigation, Resources, Writing – original draft. **Umme Sadia Salsabil:** Investigation, Resources, Writing – original draft. **Md. Masudul Islam:** Investigation, Writing – review & editing, Visualization.

## Declaration of competing interest

The authors declare that they have no known competing financial interests or personal relationships that could have appeared to influence the work reported in this paper.

## Data Availability

No data was used for the research described in the article.
